# A multicenter randomized controlled trial of surgery alone or surgery with atrial natriuretic peptide in lung cancer surgery: study protocol for a randomized controlled trial

**DOI:** 10.1186/s13063-017-1928-1

**Published:** 2017-04-20

**Authors:** Takashi Nojiri, Haruko Yamamoto, Toshimitsu Hamasaki, Kaori Onda, Kikuko Ohshima, Yasushi Shintani, Meinoshin Okumura, Kenji Kangawa, on behalf of the Japan Human Atrial Natriuretic Peptide for Lung Cancer Surgery (JANP Study) Group, Meinoshin Okumura, Jun Nakajima, Masahiro Tsuboi, Yasuhiro Hida, Yoshimasa Maniwa, Hiroyuki Oizumi, Masahiko Higashiyama, Yukiyasu Takeuchi, Yoshihisa Kadota, Satoshi Shiono

**Affiliations:** 1 Department of Biochemistry, National Cerebral and Cardiovascular Center Research Institute, 5-7-1, Fujishirodai, Suita-city, Osaka 565-8565 Japan; 2 Department of General Thoracic Surgery, Osaka University Graduate School of Medicine, 2-2 (L5) Yamadaoka, Suita-City, Osaka 565-0871 Japan; 3 Department of General Thoracic Surgery, National Hospital Organization Toneyama Hospital, 5-1-1 Toneyama, Toyonaka-City, Osaka 560-8552 Japan; 4 Center for Advancing Clinical and Translational Sciences, National Cerebral and Cardiovascular Center, 5-7-1, Fujishirodai, Suita-city, Osaka 565-8565 Japan; 5 Department of Data Science, National Cerebral and Cardiovascular Center, 5-7-1, Fujishirodai, Suita-city, Osaka 565-8565 Japan

**Keywords:** Lung cancer surgery, Atrial natriuretic peptide, Perioperative care, Cancer recurrence

## Abstract

**Background:**

Postoperative cancer recurrence is a major problem following curative surgery. In a previous retrospective study of lung cancer surgery, we reported that administration of atrial natriuretic peptide (ANP) during the perioperative period reduced postoperative recurrence. We demonstrated that ANP inhibited the adhesion of cancer cells to vascular endothelium as a vasoprotective action. The objective of this study is to evaluate the effects of ANP on the incidence of postoperative cancer recurrence in lung cancer surgery.

**Methods/design:**

The present study is a multicenter, randomized trial with two parallel groups of patients with lung cancer comparing surgery alone and surgery with ANP administration for 3 days during the perioperative period. A total of 500 patients will be enrolled from 10 Japanese institutions. The primary endpoint is 2-year relapse-free survival (RFS). The secondary endpoints are 2-year cancer-specific RFS, 5-year RFS, overall survival, the incidence of postoperative complications, and the completion rate of ANP treatment.

**Discussion:**

The principal question addressed in this trial is whether ANP with its vasoprotective action can reduce cancer recurrence following lung cancer surgery.

**Trial registration:**

UMIN Clinical Trials Registry identifier: UMIN000018480. Registered on 31 July 2015.

**Electronic supplementary material:**

The online version of this article (doi:10.1186/s13063-017-1928-1) contains supplementary material, which is available to authorized users.

## Background

Cancer recurrence following curative surgery is a major problem after most cancer treatments. More than 50% of patients with resectable non-small cell lung cancer (NSCLC) will have recurrence after curative surgery [1]. Although complete surgical resection is currently considered the best option for cure in patients with most solid tumors, it is possible that surgical trauma itself influences the development of early recurrence [2–4]. First, handling of the tumor during resection can provoke detachment of tumor cells, and the amount of circulating tumor cells is enhanced during resection of the primary tumor [5, 6]. We previously reported that the presence of circulating tumor cells in the pulmonary vein during lung cancer surgery could be a prognostic indicator for early cancer recurrence [7]. Second, surgical trauma provokes a severe inflammatory reaction. There is emerging evidence to suggest that systemic inflammation can accelerate the adhesion of circulating tumor cells to the vascular endothelium of distant organs, which is a first step during extravasation in hematogenous metastasis [8, 9]. Although most circulating tumor cells disappear rapidly [10], it is possible that residual cancer cells could attach more efficiently to the vascular endothelium by the release of inflammatory cytokines during the perioperative period [8, 9]. Because no prophylactic strategy for cancer recurrence during the perioperative period has been established, the development of effective treatment is desirable.

We identified human atrial natriuretic peptide (ANP) as a diuretic/natriuretic and vasodilating hormone in 1984 [11]. ANP binds specifically to the guanylyl cyclase A receptor and has biological functions, including diuresis and inhibition of the renin-angiotensin-aldosterone system, as well as anti-inflammatory and antifibrotic actions [12, 13]. In the clinical setting, ANP has been used to treat patients with acute heart failure in Japan since 1995. We have previously reported that the perioperative administration of ANP had a prophylactic effect on postoperative cardiopulmonary complications by attenuating the operation-induced inflammation in patients who underwent curative surgery for lung cancer [14–16]. Recently, we showed that cancer recurrence after curative surgery was significantly lower in ANP-treated patients than in control patients (surgery alone) [17]. We found that ANP inhibited the adhesion of cancer cells to vascular endothelium by suppressing E-selectin expression induced by inflammation [17]. On the basis of our previous findings, we planned a multicenter, phase II randomized controlled trial to confirm the effects of ANP on preventing postoperative recurrence following lung cancer surgery.

## Methods/design

### Purpose

The purpose of the present study is to evaluate the effects of ANP on reducing postoperative cancer recurrence in patients with lung cancer undergoing curative surgery.

### Study setting

**Fig. 1:**
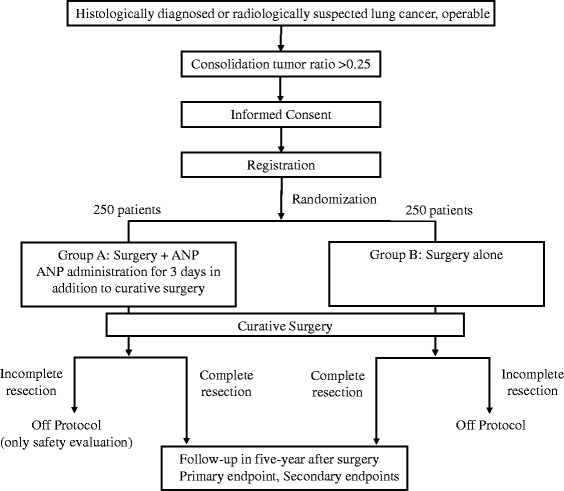
Consolidated Standards of Reporting Trials (CONSORT) flowchart. *ANP* Atrial natriuretic peptide

The study is an investigator-initiated, multi-institutional, two-arm, open-label, phase II randomized trial. The flowchart of the trial is shown in Fig. 1. The Standard Protocol Items: Recommendations for Interventional Trials (SPIRIT) 2013 checklist is given in Additional file 1.

### Endpoints

The primary endpoint of this study is the 2-year relapse-free survival (RFS) rate after curative surgery for lung cancer. The secondary endpoints are 2-year cancer-specific RFS, 5-year RFS, 5-year overall survival, the rate of patients who complete ANP treatment, and the incidence of postoperative complications.

### Patient selection

The tumors are staged according to the seventh edition of the Union for International Cancer Control *TNM Classification of Malignant Tumours* [18, 19]. Inclusion criteria are as follows:

1. NSCLC is suspected.
2. Radiologically diagnosed invasive lung tumor with a consolidation/tumor ratio >0.25 is visualized by thin-section computed tomography (CT).
3. Complete resection including mediastinal lymph node dissection is planned.
4. Patients must not have synchronous or metachronous (within 5 years) malignancies, except for carcinoma in situ or mucosal tumors curatively treated with local therapy.
5. Patients must be aged 20 years or older.
6. Eastern Cooperative Oncology Group performance status must be 0–2.
7. Organ function must be sufficient (leukocyte count ≥1500/ml, platelet count ≥1.0 × 10^5^/ml, hemoglobin ≥8.0 g/dl, total bilirubin ≤1.5 mg/dl, aspartate aminotransferase ≤100 IU/L, alanine aminotransferase ≤100 IU/L, peripheral arterial oxygen saturation on room air ≥92%).
8. Written informed consent is provided by the patient.

### Exclusion criteria

The exclusion criteria are as follows:

1. Dominant pure ground-glass opacity (GGO) lesion (radiological noninvasive lung tumor with consolidation/tumor ratio ≤0.25 visualized by thin-section CT)
2. Active concurrent malignant diseases
3. Pregnant, lactating, or potentially pregnant
4. Mental disorders that may affect the ability or willingness to provide informed consent or abide by the study protocol
5. Systemic steroids or immunosuppressive agent medication
6. Uncontrollable infectious disease, autoimmune disease, or other severe comorbidities
7. History of right ventricular infarction
8. Severe hypotension
9. Inappropriate for enrollment based on the judgment of the investigator

### Registration

Eligible patients are registered and randomly assigned to either the surgery with ANP group or the surgery-alone group by the covariate-adaptive randomization method (Pocock-Simon procedure) including sex (male/female), age (<70/≥70 years), clinical stage (IA/IB/≥II), CT findings (including GGO/not including GGO), and institution as covariates and strata. The randomization ratio is 1:1. Both patients and investigators are open to treatment allocation. The Clinical Study Data Collecting System is used for patient registration and randomization, and the Research Electronic Data Capture (REDCap™) system is used for data management. Enrollment was started in September 2015 and scheduled to continue for 2 years.

### Treatment methods

Treatment flow is shown in Fig. 1. The patients enrolled in this study receive surgery alone or surgery with ANP (group A, curative surgery with ANP; group B, curative surgery alone).

In both groups, the surgical procedures undertaken include segmentectomy, lobectomy, or pneumonectomy with systematic node dissection in open thoracotomy or video-assisted thoracic surgery. Standard systematic node dissection (ND2) includes complete removal of the hilar and mediastinal nodes.

In group A, human ANP (Daiichi-Sankyo Pharmaceutical Inc., Tokyo, Japan) is continuously infused intravenously at 0.025 μg∙kg^−1^∙minute^−1^ for 72 h beginning more than 2 h before the start of surgery. In group B, there is no agent used in addition to curative surgery. The protocol treatment is to be stopped if curative surgery is not performed.

### Follow-up

**Fig. 2:**
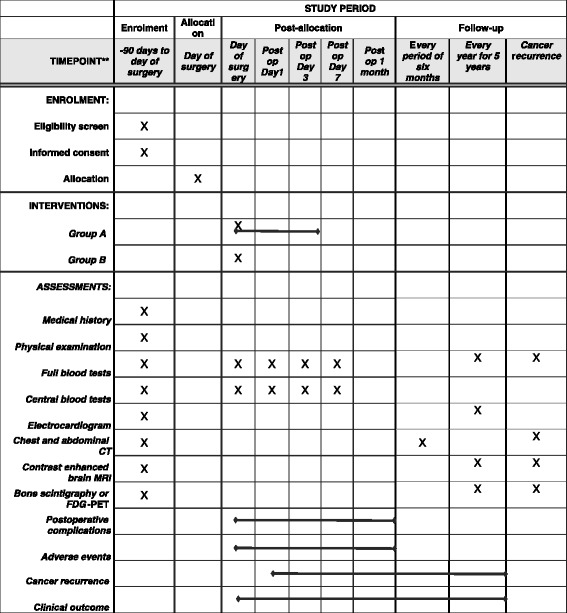
Schedule of enrollment, interventions, and assessments for the Japan Human Atrial Natriuretic Peptide for Lung Cancer Surgery Study. *CT* Computed tomography, *FDG-PET* Fluorodeoxyglucose-positron emission tomography, *MRI* Magnetic resonance imaging

After curative resection, the information regarding postoperative complications within 30 days following surgery is recorded and sent to the data center. The incidence of postoperative complications is one of the secondary endpoints. All patients are followed with scheduled examinations, including a physical examination, serum biochemistry testing, chest x-ray, contrast-enhanced chest and abdominal CT, contrast-enhanced brain magnetic resonance imaging (MRI), and bone scintigraphy to detect postoperative recurrence for 5 years. Fluorodeoxyglucose-positron emission tomography (FDG-PET) can replace bone scintigraphy. Chest and abdominal CT are performed every 6 months following surgery. Brain MRI and bone scintigraphy or FDG-PET are performed every year following surgery. The schedule of this trial is shown in Fig. 2.

### Radiological assessment

Radiographic reviews for the development of postoperative recurrence are performed by the independent central image reading board, which consists of three certified radio-oncologists. All board members are blinded to treatment allocation. All films, including chest and abdominal CT, brain MRI, and bone scintigraphy or FDG-PET scans, are reviewed regularly by the board.

### Statistical methods

The present study is a phase II randomized trial to evaluate the efficacy and safety of ANP administration during lung cancer surgery. This study is designed primarily to evaluate the 2-year RFS in both groups and to demonstrate whether ANP prevents postoperative recurrence within 2 years after curative surgery. In the previous study [17], the hazard ratio for the 2-year RFS was estimated to be 0.28 (95% confidence interval 0.09–0.87) in curative surgery with ANP compared with curative surgery alone. Because this previous observational study was retrospective, we conservatively assumed the hazard ratio to be 0.50 in the present study as the upper limit of the 70% confidence interval of the previous study. To evaluate the superiority of curative surgery with ANP (group A) over curative surgery alone (group B), the total sample size was calculated as 409 patients (95 events in total are expected), assuming 70% RFS in group B with 90% power at a one-sided αlevel of 2.5% using a log-rank test, assuming an expected accrual period of 2 years and a follow-up period of 2 years. A total of 500 patients will be recruited into the study, accounting for patients lost to follow-up, such as for benign tumors or incomplete resection.

Analyses will be done on the basis of the full analysis set (FAS). Based on the intention-to-treat (ITT) principle, the FAS includes all randomized subjects without major protocol violations. An ITT-based or per-protocol based analysis will be done to assess the robustness of the conclusions derived from the FAS-based analysis. Patient demographic data will be analyzed descriptively; categorical variables will be assessed with the chi-square test or Fisher’s exact test, whereas continuous variables will be assessed with Student’s *t* test or the Wilcoxon rank-sum test, as appropriate. The survival curves for 2-year RFS (primary endpoint) and the 2-year cancer-specific RFS, 5-year RFS, and overall survival (secondary endpoints) will be estimated using the Kaplan-Meier method and compared between the two groups by the log-rank test. The hazard ratio with its 95% confidence interval will be calculated using a proportional hazards model. The proportional hazards assumption will be investigated graphically, with a test based on Schoenfeld residuals. Safety data will be analyzed descriptively for the treated set, which consists of all randomized patients who receive at least one study treatment. All reported *P* values will be two-sided. The statistical analysis plan, which includes more technical and detailed elaboration of the principal features stated in the protocol, will be prepared separately and finalized before database-locking. All statistical analyses will be conducted at the data center in the National Cerebral and Cardiovascular Research Center.

### Centralized monitoring and the Data and Safety Monitoring Committee

Centralized monitoring is conducted for early identification and mitigation of data quality risk issues that may compromise the validity of the study results and for better assessment of patient safety. Also, the Data and Safety Monitoring Committee (DSMC) will independently review the report of trial monitoring regarding the efficacy and safety data derived from this study. On the basis of monitoring, the DSMC could consider early termination of a treatment regimen if a treatment-related death were to occur in group A during enrollment. The protocol compliance, safety, and on-schedule study progress are also monitored by the DSMC.

### Participating institutions

The participating institutions in the study are Osaka University Hospital, The University of Tokyo Hospital, Hokkaido University Hospital, National Cancer Center Hospital East, Yamagata University Hospital, Kobe University Hospital, National Hospital Organization Toneyama Hospital, Osaka Medical Center for Cancer and Cardiovascular Diseases, Osaka Prefectural Medical Center for Respiratory and Allergic Diseases, and Yamagata Prefectural Central Hospital.

## Discussion

To the best of our knowledge, this study is the first multicenter randomized clinical trial to examine the vasoprotective effects of ANP on cancer recurrence following curative surgery in patients with lung cancer. Surgical procedures may induce postoperative complications in the acute phase and cancer recurrence in the chronic phase after surgery by releasing inflammatory cytokines [2, 3]. Vascular endothelial cells that become inflamed during the perioperative period can easily adhere to immune cells or circulating tumor cells, resulting in postoperative complications or cancer recurrence [8, 9]. ANP has an anti-inflammatory action by reducing E-selectin levels in vascular endothelial cells [17]. In this study, we will be able to prospectively evaluate the effects of ANP on preventing both postoperative complications in the acute phase and cancer recurrence in the chronic phase after surgery. Surgical specimen and blood samples during the perioperative period will be collected to evaluate the mechanism of the effects of ANP, including serum soluble E-selectin levels.

Because the target of ANP is considered to be the vascular endothelium, this treatment can be used for all types of cancer surgery in principle. Most chemotherapy cannot be used during the perioperative period to reduce the incidence of cancer recurrence, owing to cytotoxicity. However, ANP has been proven not to cause severe adverse effects or hypotension on the basis of its use in patients with acute heart failure in Japan for over 20 years [20]. The results of this study will provide clinically valuable information for future cancer treatment.

### Trial status

We are in the process of recruiting patients.

## Additional file

Additional file 1: SPIRIT 2013 checklist: recommended items to address in a clinical trial protocol and related documents. (DOC 121 kb)

## Abbreviations

ANP: Atrial natriuretic peptide
CONSORT: Consolidated Standards of Reporting Trials
CT: Computed tomography
DSMC: Data and Safety Monitoring Committee
FAS: Full analysis set
FDG-PET: Fluorodeoxyglucose-positron emission tomography
GGO: Ground-glass opacity
ITT: Intention to treat
JANP: Japan Human Atrial Natriuretic Peptide for Lung Cancer Surgery Study
MRI: Magnetic resonance imaging
ND2: Standard systematic node dissection
NSCLC: Non-small cell lung cancer
REDCap: Research Electronic Data Capture
RFS: Relapse-free survival
SPIRIT: Standard Protocol Items: Recommendations for Interventional Trials
